# Microscopically detectable maternal schistosomiasis at delivery in Eastern Uganda: Prevalence, microscopy-based species distribution and associated household WASH characteristics in a hospital-based cross-sectional study

**DOI:** 10.1371/journal.pgph.0006297

**Published:** 2026-10-23

**Authors:** George Binna Sande, Musa Kasujja, Emmanuel Okurut, Maxwell Okello, Marie Pascaline Sabine Ishimwe, Theodore Nteziyaremye, John Yaweh, Daniel Mugabi, Sara Liban, Geoffrey Okot, G. R. Neel, Bot Yakubu Sunday, Matthew Chibunna Igwe, Amos Muhumuza, Theoneste Hakizimana

**Affiliations:** 1 Department of Obstetrics and Gynecology, Kampala International University, Ishaka, Uganda; 2 Department of Pediatrics and Child Health, Kampala International University, Ishaka, Uganda; 3 Department of Sciences, University of Rwanda, Kigali, Rwanda; 4 Department of Microbiology, Kampala International University, Ishaka, Uganda; 5 Department of Medical Laboratory Sciences, Kampala International University, Ishaka, Uganda; 6 Department of Public Health, Kampala International University, Ishaka, Uganda; Southern Medical University, CHINA

## Abstract

Schistosomiasis remains an important but neglected public health problem in Uganda. We estimated its prevalence, microscopy-based species distribution and associated factors among women delivering at Jinja Regional Referral Hospital. A hospital-based analytical cross-sectional study included 392 women from 1 September to 30 December 2025. One stool and one urine specimen per participant were examined microscopically for Schistosoma mansoni and S. haematobium, respectively. Multivariable logistic regression assessed associated factors; parsimonious conventional and Firth penalized models were used in sensitivity analyses. Microscopically detectable schistosomiasis was present in 44/392 women (11.2%; 95% CI: 8.5–14.8). Among microscopy-positive women, S. haematobium accounted for 63.6% of infections (95% CI: 48.2–76.7), S. mansoni for 29.5% (95% CI: 17.7–45.0), and mixed infection for 6.8% (95% CI: 2.1–19.6). Unimproved main water source (adjusted odds ratio [aOR] = 3.11; 95% CI: 1.46–6.64; p = 0.003), unimproved toilet facility (aOR = 4.40; 95% CI: 2.01–9.67; p < 0.001) and absence of a handwashing facility (aOR = 2.12; 95% CI: 1.02–4.41; p = 0.043) remained associated with microscopy-detected infection after adjustment. Women reporting fewer than four antenatal care contacts had higher adjusted odds than those reporting four to seven contacts (aOR = 8.43; 95% CI: 3.21–22.18; p < 0.001). The principal WASH associations remained consistent in sensitivity analyses. S. haematobium predominated among microscopy-classified infections. Household WASH characteristics remained associated with microscopy-detected infection, while the antenatal care association requires cautious interpretation. These cross-sectional findings do not establish causality but support further evaluation of context-appropriate schistosomiasis risk assessment within maternal health services and coordinated WASH and schistosomiasis-control strategies in endemic settings.

## Introduction

Schistosomiasis is a neglected tropical disease caused by trematode parasites of the genus *Schistosoma* and remains a major public health problem in many low- and middle-income countries, particularly in sub-Saharan Africa [1,2]. Transmission occurs when people come into contact with freshwater contaminated with cercariae released by infected snail intermediate hosts, making inadequate water, sanitation, and hygiene conditions central to ongoing transmission [3,4]. More than 90% of the global burden of schistosomiasis occurs in sub-Saharan Africa, where the disease continues to affect socially and economically vulnerable populations with limited access to safe water and adequate sanitation [1,5]

Women of reproductive age are an important but relatively under-studied population in schistosomiasis research. An estimated 40 million women of childbearing age are affected globally, yet the epidemiology of schistosomiasis during pregnancy and around delivery remains insufficiently characterized in many endemic settings [6]. A systematic review of studies from Africa reported a pooled prevalence of schistosomiasis in pregnancy of 13.2% (95% CI 11.0–15.4), highlighting a substantial burden across the continent [3]. In another global systematic review, pooled prevalence estimates among pregnant women were 13.44% for *Schistosoma haematobium* and 12.18% for *Schistosoma mansoni*, while *S. japonicum* was reported mainly outside Africa [6]. Individual studies have reported even higher burdens in some endemic settings, including 31.9% in Cameroon and 40.4% in Madagascar, demonstrating marked geographic heterogeneity and the need for local epidemiologic data [7,8]

Understanding species distribution is particularly important in endemic African settings because the two major human species, *S. haematobium* and *S. mansoni*, differ in clinical manifestations, transmission ecology, and control implications [5,9]. In Africa, *S. haematobium* and *S. mansoni* are the predominant species infecting pregnant women, with *S. haematobium* reported more frequently than *S. mansoni* in meta-analytic estimates from African studies [3,6]. Uganda is endemic for schistosomiasis, and both intestinal and urogenital forms are present, with intestinal schistosomiasis concentrated along Lake Victoria, Lake Albert, and the Nile, and urogenital schistosomiasis more common in parts of central Uganda [5,9]. A national survey reported an overall schistosomiasis prevalence of 25.6% in the general population, underscoring the persistent burden of the disease in Uganda [9]. However, data specific to pregnant women and women delivering in Ugandan hospital settings remain scarce

Evidence from endemic settings suggests that schistosomiasis in pregnant women is shaped by environmental and social determinants, especially those related to water contact and sanitation [10]. In rural Zimbabwe, maternal age, education, marital status, religion, household drinking water source, latrine access, study region, and season were associated with urogenital schistosomiasis among pregnant women [11]. In Cameroon, younger age, residence, blood group O, and lower education level were associated with maternal schistosomiasis, although some obstetric and behavioral variables were not significant [8]. A broader African review also identified proximity to water bodies as an important driver of infection risk [12]. These findings suggest that local transmission patterns are context-specific and that site-level data are essential for designing effective screening and prevention strategies.

In Uganda, most schistosomiasis research and control activities have focused on school-aged children, general populations and highly endemic lakeshore communities. The limited studies involving mothers have largely been conducted in small, high-transmission communities around major lakes and have predominantly examined intestinal schistosomiasis. These findings may not reflect women accessing hospital-based delivery services in eastern Uganda, where obstetric patients originate from urban, peri-urban and rural communities with heterogeneous freshwater exposure and household WASH conditions. Additionally, Jinja Regional Referral Hospital serves a population in eastern Uganda living within a schistosomiasis-endemic ecological zone influenced by Lake Victoria and surrounding freshwater exposure.

Delivery represents an important but underused point of contact for identifying neglected infections because women who may have had inconsistent antenatal attendance can be reached through maternity services. However, the prevalence of microscopically detectable schistosomiasis, distribution of Schistosoma species and household WASH conditions associated with infection among women delivering at referral hospitals in eastern Uganda remain poorly characterized. We therefore determined the prevalence of microscopically detectable schistosomiasis, described the microscopy-based species distribution among microscopy-positive women, and identified factors associated with microscopically detectable infection among women delivering at Jinja Regional Referral Hospital.

## Materials and methods

### Study design and settings

This hospital-based cross-sectional analytical study was conducted in the postnatal ward of Jinja Regional Referral Hospital (JRRH) from 1^st^ September 2025–30^th^ December 2025 among postpartum women delivering at the hospital. The design enabled estimation of the prevalence of microscopically detectable schistosomiasis, description of the microscopy-based species distribution among microscopy-positive women, and assessment of factors associated with microscopically detectable infection. Jinja Regional Referral Hospital is a public tertiary referral hospital serving an estimated catchment population of approximately 4.6 million people across Jinja City and 11 surrounding districts. It receives obstetric referrals from district hospitals and lower-level health facilities within and beyond the Busoga sub-region. Consequently, women delivering at the hospital include residents of Jinja City and women referred from surrounding rural and peri-urban communities. The study population therefore represents women accessing delivery services at this regional referral hospital rather than all pregnant women in eastern Uganda.

### Study population

The study population consisted of postpartum women who delivered at Jinja Regional Referral Hospital and were admitted to the postnatal ward during the study period.

### Inclusion criteria

Women were eligible for inclusion if they had delivered at Jinja Regional Referral Hospital during the study period and provided written informed consent to participate.

### Exclusion criteria

Women who were severely ill or unable to provide the required information or specimens at the time of data collection were excluded from the study.

### Sample size determination

The sample size was determined using the Kish Leslie formula:


$$\begin{array}{c} N=\frac{Z^{\text{2}}p(\text{1}-p)}{d^{\text{2}}} \end{array}$$

Where n was the minimum required sample size, Z = 1.96 corresponded to a 95% confidence level, p = 0.62 was the anticipated prevalence, and d = 0.05 was the desired absolute precision. The anticipated prevalence was obtained from a longitudinal study conducted in a high-transmission community along Lake Albert, Uganda, which reported a baseline prevalence of intestinal Schistosoma mansoni infection of 62% among 37 mothers (5). This estimate was selected because no published prevalence estimate specifically among women delivering at JRRH or a comparable Ugandan referral hospital was available during protocol development. The estimate was used as an a priori planning parameter and was not considered representative of postpartum women in Jinja.


$$\begin{array}{c} \text{n }=\frac{{\text{1}.\text{96}}^{\text{2}}\text{x 0}.\text{62 x }(\text{1}\;-\;\text{0}.\text{62})}{{\text{0}.\text{05}}^{\text{2}}}\;=\text{ 362}.\text{03} \end{array}$$

The minimum sample size was rounded up to 363 participants. No separate allowance for nonresponse or incomplete specimens was added to this minimum. During recruitment, 420 women were assessed for eligibility, and 392 women with complete questionnaire, stool-microscopy and urine-microscopy results were included in the final analysis, exceeding the calculated minimum by approximately 8%. The final sample also exceeded the approximately 384 participants required under a 50% prevalence assumption, which provides the maximum-variability benchmark for this formula.

### Sampling technique

Systematic random sampling was applied prospectively throughout the three-month recruitment period. Women presenting for delivery were considered sequentially in their order of presentation. The sampling interval, calculated from the anticipated number of deliveries and the required sample size, was approximately two. After randomly selecting the first woman from the first two presenting women, every second woman was selected for eligibility assessment. If a selected woman was ineligible or declined participation, this was recorded and the established sampling sequence continued without restarting the interval.

### Data collection procedure

Eligible postpartum women were approached in the postnatal ward, provided with detailed information about the study, and enrolled after giving written informed consent in English or the local language. Data were collected using a structured interviewer-administered questionnaire capturing socio-demographic characteristics, water, sanitation and hygiene practices, and obstetric/medical history. Stool and urine samples were subsequently collected and processed using standard procedures for microscopic diagnosis of *Schistosoma mansoni* and S. haematobium. Microscopy-positive women were informed of their results and referred for appropriate care.

### Study variables

The independent variables in this study included socio-demographic, behavioral, and medical/obstetric factors that were considered to be associated with schistosomiasis among women delivering at Jinja Regional Referral Hospital. These included age, marital status, occupation, residence, employment status, education level, and income level, main source of water, handwashing practices, type of toilet facility used, antenatal care attendance, HIV status, and body mass index. The dependent variable was maternal schistosomiasis infection, determined by stool microscopy for *Schistosoma mansoni* and urine microscopy for S. haematobium. For participants diagnosed with schistosomiasis, the infecting species were further classified as S. haematobium, S. mansoni, or mixed infection. The primary study outcomes were therefore the prevalence of microscopically detectable schistosomiasis, species distribution among infected women, and factors associated with infection. The WASH variables assessed participants’ usual household water source, toilet facility and availability of a handwashing facility before admission for delivery. They did not refer to facilities available at JRRH. Antenatal-care attendance was categorized a priori as fewer than four, four to seven, and eight or more contacts. Four to seven contacts, the largest category, was used as the reference group to provide a statistically more stable basis for comparison. ANC attendance was analysed categorically because a linear relationship with the log odds of microscopically detectable schistosomiasis could not be assumed. Estimates for the category of eight or more contacts were interpreted cautiously because this category contained only 17 women and two microscopy-positive outcomes.

### Microscopy-based diagnosis of schistosomiasis

Each participant provided one fresh stool specimen and one terminal-stream urine specimen in separate, clean, labelled, leak-proof containers. Stool specimens were examined for Schistosoma mansoni eggs using the Kato–Katz thick-smear technique. Two slides were prepared from each specimen using a 41.7-mg template and examined microscopically for characteristic lateral-spined S. mansoni eggs.

Urine specimens were collected between 10:00 and 14:00 hours, when urinary egg excretion is generally highest. After thorough mixing, 10 mL of urine was filtered through a 12-µm polycarbonate membrane. The membrane was transferred to a glass slide and examined microscopically for characteristic terminal-spined S. haematobium eggs. One filtration slide was examined per participant.

All preparations were examined by trained laboratory technologists experienced in parasitological microscopy and working according to the hospital laboratory’s standard operating procedures. A participant was classified as microscopy-positive when characteristic Schistosoma eggs were detected in at least one stool or urine preparation. Detection of characteristic S. mansoni eggs in stool alone was classified as intestinal schistosomiasis, detection of characteristic S. haematobium eggs in urine alone was classified as urogenital schistosomiasis, and detection in both specimens was classified as mixed infection.

Preparations containing eggs that could not be confidently classified by the initial microscopist were independently examined by a second experienced laboratory technologist. Disagreements were resolved through joint review, and the agreed classification was recorded. No microscopy-positive specimen remained indeterminate after this review

### Quality control

To ensure the quality and reliability of the study findings, quality control measures were applied throughout both the data collection and laboratory processes. Before the main data collection, the questionnaire and study procedures were pretested among postpartum women at a nearby health facility that was not included in the actual study. The purpose of the pretest was to assess the clarity, relevance, flow, and consistency of the questions. Based on the feedback obtained, minor adjustments were made to improve wording and ensure that the questions were easily understood by participants.

Research assistants were trained on the study objectives, participant recruitment, informed consent procedures, questionnaire administration, and standardized methods of data collection. Throughout the study period, the principal investigator closely supervised the data collection process and reviewed completed questionnaires on a daily basis to ensure completeness, consistency, and accuracy, while minimizing missing or incorrect information.

In the laboratory, all stool and urine samples were processed by a trained laboratory scientist following standard operating procedures. Samples were labeled using unique numerical codes to ensure proper identification while maintaining participant confidentiality. Standard procedures for sample collection, transportation, storage, and processing were carefully followed to minimize contamination and diagnostic errors.

To assess the reproducibility of the microscopy findings, a randomly selected 10% of the stool and urine microscopy preparations were independently re-examined at Lancet Laboratory, an external reference laboratory. Any discordant findings were jointly reviewed and re-evaluated by the laboratory teams, and the agreed result was used in the final analysis.

### Data management and analysis

Data were collected using coded interviewer-administered questionnaires and laboratory result forms, then securely stored in locked file boxes. Electronic data were entered into Microsoft Excel, cleaned, backed up regularly, and password-protected before being exported to STATA version 14.2 for statistical analysis. Categorical variables were summarized using frequencies and percentages, whereas continuous variables were summarized using means and standard deviations (SDs) for normally distributed data or medians and interquartile ranges (IQRs) for skewed data. Statistical significance was set at p < 0.05, and 95% confidence intervals (CIs) were reported where applicable. The prevalence of microscopically detectable schistosomiasis was calculated as the proportion of women with laboratory-confirmed schistosomiasis out of all study participants, and presented as a percentage with corresponding 95% confidence intervals. Species distribution was determined from laboratory findings among infected women. Participants with *Schistosoma haematobium* eggs detected on urine microscopy only were classified as having S. haematobium infection, those with *Schistosoma mansoni* eggs detected on stool microscopy only were classified as having S. mansoni infection, and those positive on both urine and stool microscopy were classified as having mixed infection. The proportions of S. haematobium, S. mansoni, and mixed infections were then calculated among all women with schistosomiasis.

To assess factors associated with microscopically detectable schistosomiasis, bivariate logistic regression analysis was first performed for each independent variable. Crude odds ratios (cORs), 95% confidence intervals, and p-values were reported. Variables with p < 0.20 at bivariate analysis, together with those considered biologically plausible, were included in the multivariable logistic regression model to identify factors independently associated with schistosomiasis. Adjusted odds ratios (aORs), 95% confidence intervals, and p-values were reported. A p-value < 0.05 was considered statistically significant in the final adjusted analysis. ANC attendance was analyzed using the prespecified categories of fewer than four, four to seven and eight or more contacts. Four to seven contacts, the largest category, was used as the reference group to provide a more stable basis for regression comparisons. Because the expanded multivariable model contained 13 predictor parameters relative to 44 microscopy-positive outcomes, sensitivity analyses were conducted to assess possible overfitting and sparse-data bias. A parsimonious model containing the three principal household WASH variables water source, toilet facility and handwashing-facility availability was fitted using conventional logistic regression. The expanded and parsimonious models were also refitted using Firth penalized logistic regression. Model discrimination was assessed using the area under the receiver operating characteristic curve, and overall predictive accuracy was assessed using the Brier score. Calibration was examined using the Hosmer–Lemeshow goodness-of-fit test. Bootstrap internal validation using 500 resamples was used to estimate optimism-corrected discrimination.

### Ethical considerations

Ethical approval for the study was obtained from the Kampala International University Research Ethics Committee (KIU-2025–1741). The study was subsequently registered with the Uganda National Council for Science and Technology (UNCST). Administrative permission was also obtained from Jinja Regional Referral Hospital and the in-charge of the postnatal ward before the start of data collection.

Written informed consent was obtained from all participants prior to enrolment, either in English or Lusoga, depending on the participant’s preferred language. Participation in the study was entirely voluntary, and participants were informed of their right to decline participation or withdraw from the study at any time without any effect on the healthcare services they received. Confidentiality was maintained throughout the study by using unique study identification numbers instead of participants’ names, and all study records and biological samples were securely stored and accessed only by authorized study personnel.

## Results

### Basic characteristics of the study participants

Of the 420 women assessed for eligibility, 15 were excluded and 13 declined participation. The remaining 392 women provided written informed consent, completed the questionnaire, submitted stool and urine specimens, and were included in the final analysis (Fig 1). Among the 392 women enrolled in this study, the majority were aged 20–29 years, 204 (52.0%), resided in rural areas, 206 (52.6%), and were married, 275 (70.2%). Nearly half were unemployed, 192 (49.0%), and most had attained primary education, 144 (36.7%). Regarding income, 179 (45.7%) were in the middle-income category.

**Fig 1:**
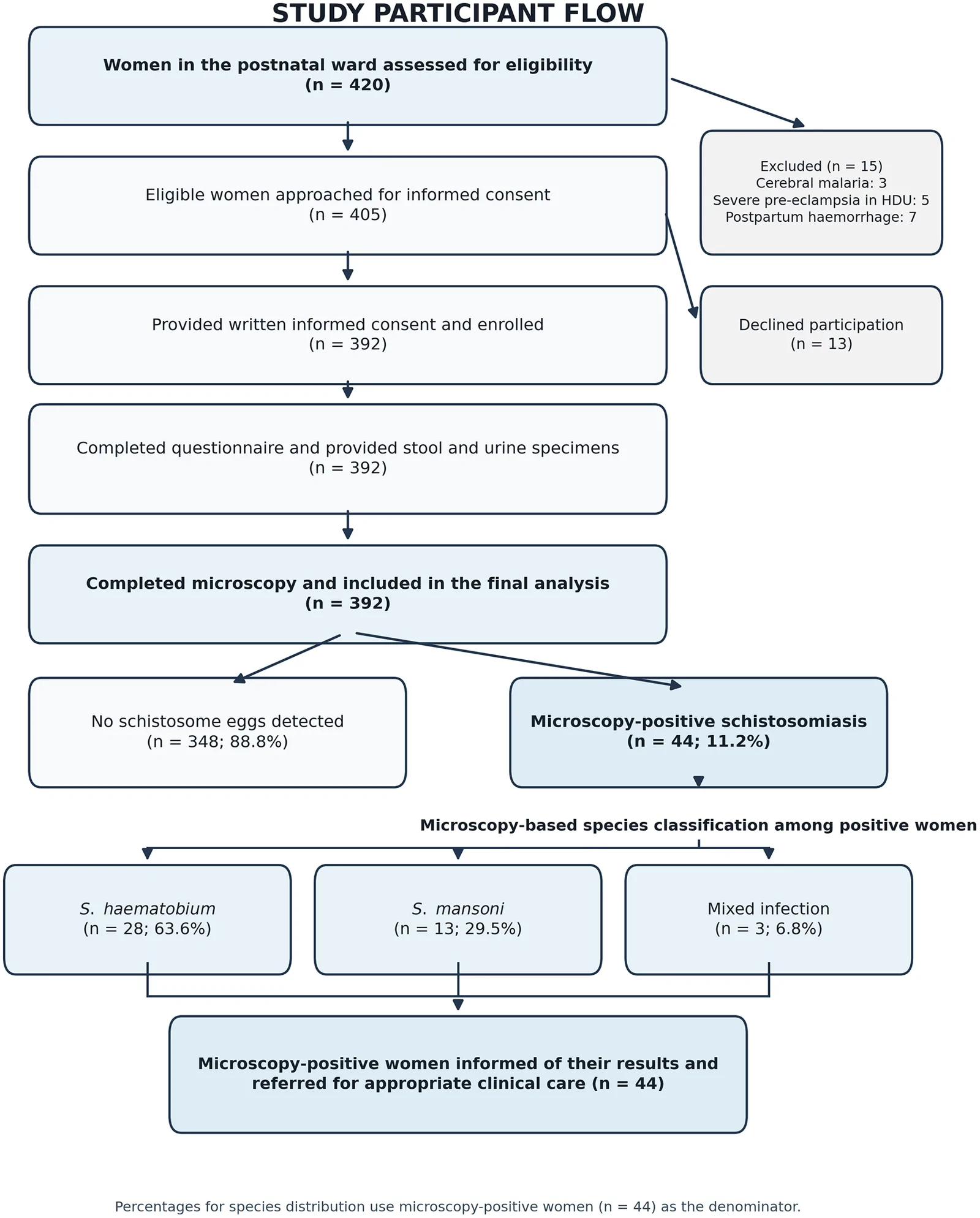
Flow of women through eligibility screening, enrolment, specimen collection, microscopy, species classification and referral for care at Jinja Regional Referral Hospital.

With respect to WASH characteristics, 190 (48.5%) used an unimproved main water source, 181 (46.2%) used an unimproved toilet facility, and 259 (66.1%) had a handwashing facility available. Nearly half of the participants had attended fewer than four ANC visits, 191 (48.7%), while 43 (11.0%) were HIV positive. Most women had normal BMI, 185 (47.2%), whereas 168 (42.9%) were overweight, 27 (6.9%) were obese, and 12 (3.0%) were underweight (Table 1). Overall, the participant profile highlights substantial social and environmental vulnerability, with nearly half of the women reporting unimproved water sources or toilet facilities, indicating that a large proportion of this maternal population was living in conditions relevant to ongoing schistosomiasis transmission.

**Table 1:** Basic characteristics of the study participants (N = 392).

Variable and categories	Total (n [%])
**Maternal age category (years)**	
<20	44 (11.2)
20-29	204 (52.0)
30-39	132 (33.7)
40+	12 (3.1)
**Residence**	
Rural	206 (52.6)
Urban	186 (47.4)
**Employment status**	
Unemployed	192 (49.0)
Informal	131 (33.4)
Formal	69 (17.6)
**Education level**	
No formal	56 (14.3)
Primary	144 (36.7)
Secondary	117 (30.0)
Tertiary	75 (19.0)
**Income level**	
Low	146 (37.2)
Middle	179 (45.7)
High	67 (17.1)
**Marital status**	
Not married	117 (29.8)
Married	275 (70.2)
**Main water source unimproved**	
No	202 (51.5)
Yes	190 (48.5)
**Toilet facility unimproved**	
No	211 (53.8)
Yes	181 (46.2)
**Handwashing facility available**	
No	133 (33.9)
Yes	259 (66.1)
**ANC visits category**	
<4	191 (48.7)
4-7	184 (47.0)
8+	17 (4.3)
**HIV status**	
Negative	349 (89.0)
Positive	43 (11.0)
**Body mass index category**	
Underweight	12 (3.0)
Normal	185 (47.2)
Overweight	168 (42.9)
Obese	27 (6.9)

Underweight (<18.5 kg/m^2^), normal weight (18.5–24.9 kg/m^2^), overweight (25.0–29.9 kg/m^2^), and obesity (≥30 kg/m^2^).

### Prevalence of microscopically detectable schistosomiasis among women delivering at Jinja regional referral hospital

Under the study’s microscopy-based diagnostic protocol, *Schistosoma* eggs were detected in 44/392 women, corresponding to a prevalence of microscopically detectable schistosomiasis of 11.2% (95% CI: 8.5–14.8) (Fig 2). Among the 44 microscopy-positive women, *Schistosoma haematobium* accounted for 28/44 detected infections (63.6%; 95% CI 48.2–76.7), *S. mansoni* for 13/44 (29.5%; 95% CI 17.7–45.0), and mixed infection for 3/44, representing 6.8% (95% CI: 2.1–19.6) (Fig 3). These proportions represent the microscopy-based species distribution among detected infections and should not be interpreted as definitive species prevalence in the entire study population.

**Fig 2:**
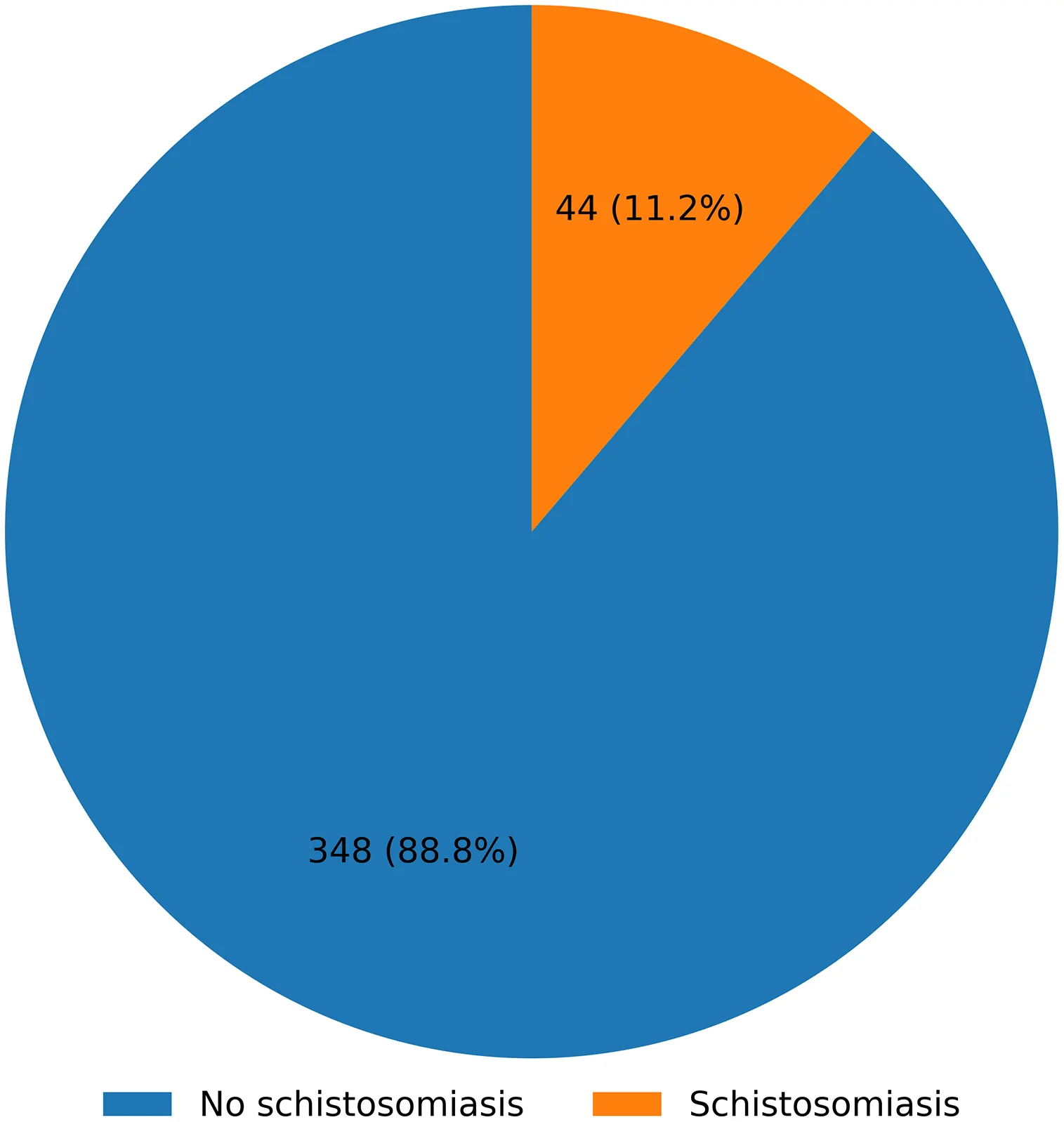
Prevalence of microscopically detectable schistosomiasis among women delivering at Jinja Regional Referral Hospital (N = 392).

**Fig 3:**
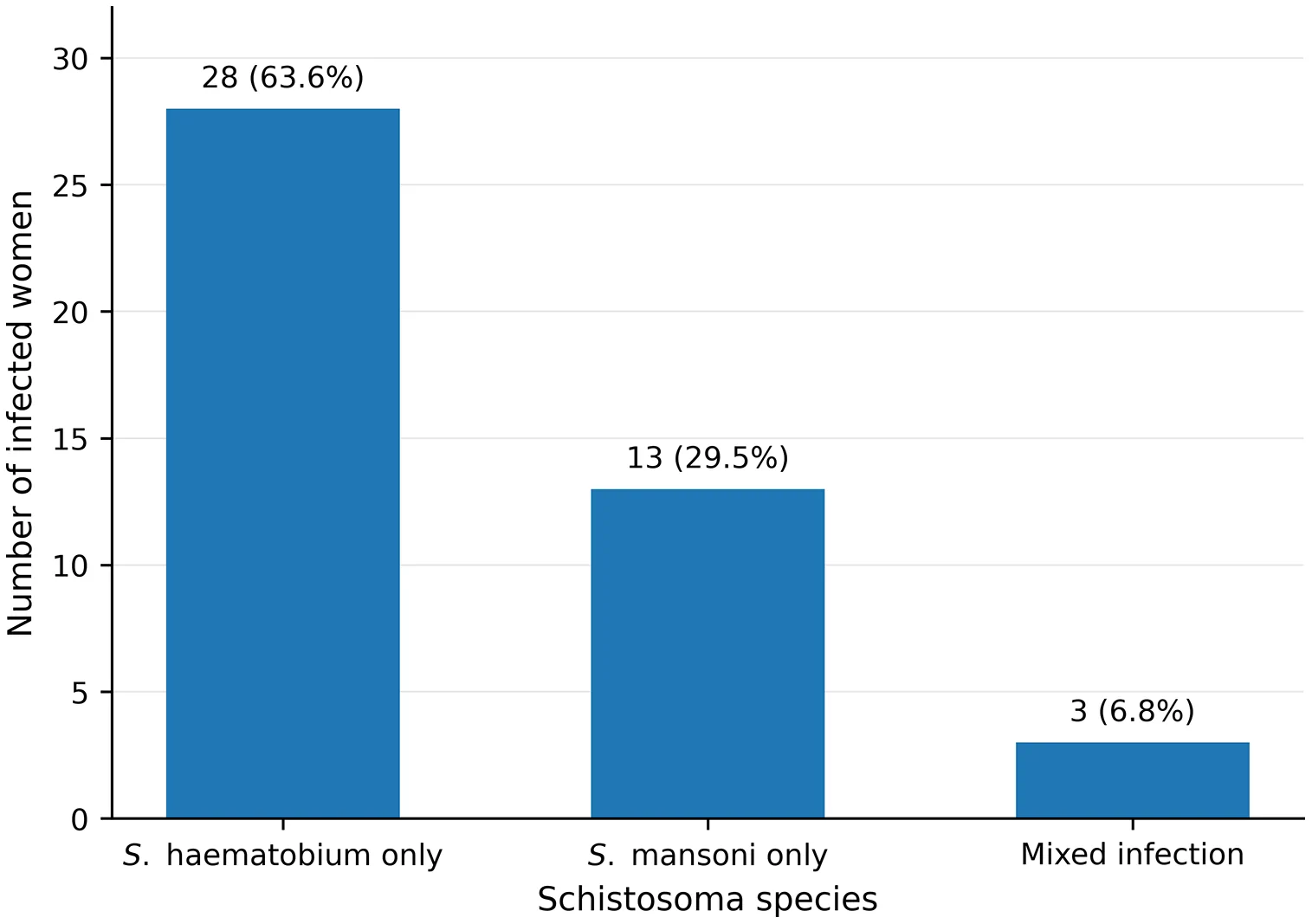
Microscopy-based Schistosoma species distribution among microscopy-positive women (n = 44).

### Factors associated with microscopically detectable schistosomiasis among women delivering at Jinja Regional Referral Hospital (N = 392)

At bivariable analysis, education level, main water source, toilet facility, handwashing-facility availability, ANC attendance and body mass index had p-values <0.20 and met the prespecified criterion for consideration in the expanded multivariable logistic regression model. Employment status was additionally retained as an indicator of socioeconomic position and a potential confounder of the associations between household WASH characteristics and microscopically detectable schistosomiasis (Table 2).

**Table 2:** Bivariable analysis of factors associated with microscopically detectable schistosomiasis among women delivering at Jinja Regional Referral Hospital (N = 392).

Variable and categories	Microscopy-negative, n (%)	Microscopy-positive, n (%)	cOR	95% CI	p- value
**Maternal age category (years)**					
<20	39 (11.2)	5 (11.4)	1.41	0.15-13.37	0.764
20-29	183 (52.6)	21 (47.7)	1.26	0.16-10.27	0.828
30-39	115 (33.0)	17 (38.6)	1.63	0.20-13.41	0.651
40+	11 (3.2)	1 (2.3)	1.00		
**Residence**					
Rural	183 (52.6)	23 (52.3)	0.99	0.53-1.85	0.969
Urban	165 (47.4)	21 (47.7)	1.00		
**Employment status**					
Unemployed	165 (47.4)	27 (61.4)	1.45	0.60-3.50	0.409
Informal	121 (34.8)	10 (22.7)	0.73	0.27-2.02	0.546
Formal	62 (17.8)	7 (15.9)	1.00		
**Education level**					
No formal	46 (13.2)	10 (22.7)	3.04	0.98-9.48	**0.055**
Primary	129 (37.1)	15 (34.1)	1.63	0.57-4.67	0.364
Secondary	103 (29.6)	14 (31.8)	1.90	0.66-5.52	0.237
Tertiary	70 (20.1)	5 (11.4)	1.00		
**Income level**					
Low	131 (37.6)	15 (34.1)	1.16	0.43-3.15	0.765
Middle	156 (44.8)	23 (52.3)	1.50	0.58-3.86	0.402
High	61 (17.5)	6 (13.6)	1.00		
**Marital status**					
Not married	101 (29.0)	16 (36.4)	1.40	0.72-2.69	0.318
Married	247 (71.0)	28 (63.6)	1.00		
**Main water source unimproved**					
Yes	161 (46.3)	29 (65.9)	2.25	1.16-4.34	**0.016**
No	187 (53.7)	15 (34.1)	1.00		
**Toilet facility unimproved**					
Yes	149 (42.8)	32 (72.7)	3.56	1.77-7.15	**<0.001**
No	199 (57.2)	12 (27.3)	1.00		
**Handwashing facility unavailable**					
No	111 (31.9)	22 (50.0)	2.14	1.13-4.02	**0.019**
Yes	237 (68.1)	22 (50.0)	1.00		
**ANC visits category**					
4-7	178 (51.1)	6 (13.6)	1.00		
<4	155 (44.5)	36 (81.8)	6.89	2.83-16.79	**<0.001**
8+	15 (4.3)	2 (4.6)	3.96	0.73-21.33	0.110
**HIV status**					
Positive	38 (10.9)	5 (11.4)	1.05	0.39-2.81	0.929
Negative	310 (89.1)	39 (88.6)	1.00		
**Body mass index category**					
Underweight	10 (2.9)	2 (4.5)	1.86	0.38-9.14	0.447
Overweight	151 (43.4)	17 (38.6)	1.04	0.52-2.10	0.903
Obese	20 (5.7)	7 (15.9)	3.25	1.21-8.73	**0.020**
Normal	167 (48.0)	18 (40.9)	1.00		

cOR, crude odds ratio; CI, confidence interval. Bold type indicates variables with P < .20. Body mass index was classified as underweight (<18.5 kg/m^2^), normal weight (18.5–24.9 kg/m^2^), overweight (25.0–29.9 kg/m^2^) or obesity (≥30 kg/m^2^). The four-to-seven-contact category was used as the reference group because it was the largest category and provided a statistically more stable comparison than the category of eight or more contacts, which contained only 17 women and two microscopy-positive outcomes. Percentages in the microscopy-negative and microscopy-positive columns were calculated using their respective outcome-column totals as denominators and therefore do not represent the prevalence of microscopically detectable schistosomiasis within each exposure category.

Using 4–7 ANC contacts as the reference category, women reporting fewer than four contacts had higher crude odds of microscopically detectable schistosomiasis (cOR 6.89, 95% CI 2.83–16.79; p < 0.001). The crude estimate for women reporting eight or more contacts was imprecise and did not provide clear evidence of a difference from the reference category (cOR 3.96, 95% CI 0.73–21.33; p = 0.110).

After adjustment for the covariates included in the expanded model, three household WASH characteristics remained associated with microscopically detectable schistosomiasis. Women using an unimproved toilet facility had higher adjusted odds than those using an improved facility (aOR 4.40, 95% CI 2.01–9.67). Higher adjusted odds were also observed among women using an unimproved main water source (aOR 3.11, 95% CI 1.46–6.64) and those without an available handwashing facility (aOR 2.12, 95% CI 1.02–4.41), compared with their respective reference groups. Women reporting fewer than four ANC contacts also had higher adjusted odds than those reporting 4–7 contacts (aOR 8.43, 95% CI 3.21–22.18; p < 0.001). The estimate for women reporting eight or more contacts remained imprecise and did not provide clear evidence of a difference from the reference category (aOR 4.13, 95% CI 0.67–25.43; p = 0.126) (Table 3). These ANC findings should be interpreted cautiously because attendance may reflect differences in healthcare access, socioeconomic circumstances and other unmeasured characteristics rather than a direct effect on schistosomiasis. The sensitivity analyses supported the findings of the expanded conventional logistic regression model. Unimproved water sources, unimproved toilet facilities and lack of a handwashing facility remained associated with microscopically detectable schistosomiasis in both the parsimonious conventional and Firth penalized models (S1 Table). The expanded conventional model showed good discrimination, with an apparent AUC of 0.844, and a Brier score of 0.081. The Hosmer–Lemeshow test showed no strong evidence of poor calibration (p = 0.065). After bootstrap internal validation, the optimism-corrected AUC was 0.790. Further results from the sensitivity analyses and model-adequacy assessments are presented in S1 Table.

**Table 3:** Multivariable logistic regression analysis of factors associated with microscopically detectable schistosomiasis among women delivering at Jinja Regional Referral Hospital (N = 392).

Variable and categories	cOR	95% CI	p-value	aOR	95% CI	p-value
**Employment status**						
Unemployed	1.45	0.60-3.50	0.409	1.71	0.64-4.59	0.283
Informal	0.73	0.27-2.02	0.546	0.55	0.18-1.71	0.303
Formal	1.00			1.00		
**Education level**						
No formal	3.04	0.98-9.48	0.055	3.45	0.95-12.52	0.059
Primary	1.63	0.57-4.67	0.364	1.72	0.52-5.67	0.375
Secondary	1.90	0.66-5.52	0.237	1.65	0.50-5.48	0.413
Tertiary	1.00			1.00		
**Main water source unimproved**						
Yes	2.25	1.16-4.34	0.016	3.11	1.46-6.64	**0.003**
No	1.00			1.00		
**Toilet facility unimproved**						
Yes	3.56	1.77-7.15	<0.001	4.40	2.01-9.67	**<0.001**
No	1.00			1.00		
**Handwashing facility available**						
No	2.14	1.13-4.02	0.019	2.12	1.02-4.41	**0.043**
Yes	1.00			1.00		
**ANC visits category**						
4-7	1.00			1.00		
<4	6.89	2.83-16.79	<0.001	8.43	3.21-22.18	<0.001
8+	3.96	0.73-21.33	0.110	4.13	0.67-25.43	0.126
**Body mass index category**						
Underweight	1.86	0.38-9.14	0.447	5.46	0.89-33.54	0.067
Overweight	1.04	0.52-2.10	0.903	0.79	0.36-1.72	0.548
Obese	3.25	1.21-8.73	0.020	2.73	0.85-8.73	0.091
Normal	1.00			1.00		

cOR, crude odds ratio; aOR, adjusted odds ratio; CI, confidence interval. Bold type indicates P < .05. Body mass index was classified as underweight (<18.5 kg/m^2^), normal weight (18.5–24.9 kg/m^2^), overweight (25.0–29.9 kg/m^2^) or obesity (≥30 kg/m^2^). Four to seven ANC contacts constituted the reference category.

## Discussion

The prevalence of microscopically detectable schistosomiasis in this study was 11.2%, showing that more than one in ten women presenting for delivery had a microscopy-detected infection. This prevalence is close to the pooled African estimate of 13.2% reported in a systematic review and meta-analysis among pregnant women, suggesting that the burden observed at JRRH is comparable to that reported in other endemic African maternal populations [3].

Our prevalence was lower than the Ugandan national estimate of 25.6% reported by Exum et al [9], as well as estimates reported from highly endemic Ugandan lakeshore settings [5,13] and studies among pregnant women in Cameroon, Madagascar and Zimbabwe [7,8,11]. These differences should be interpreted cautiously because they may reflect variations in study populations, local transmission intensity, freshwater exposure and diagnostic methods. Exum et al. used POC-CCA, whereas our study used stool and urine microscopy. Microscopy may miss low-intensity infections; therefore, the prevalence observed at JRRH may underestimate the underlying burden [9,14,15].

Among microscopy-positive women, *S. haematobium* accounted for 63.6% of infections, *S. mansoni* for 29.5% and mixed infection for 6.8%. This indicates that urogenital schistosomiasis was the predominant microscopy-detected form in this maternal population. The finding is consistent with broader African evidence showing that *S. haematobium* is frequently more common than *S. mansoni* among pregnant women and women of reproductive age [3,6].

Species distribution is not uniform across settings because it is influenced by local ecology, intermediate snail-host distribution and freshwater-contact patterns [16,17]. For example, *S. mansoni* predominated among pregnant women in Njombe-Penja, Cameroon, although *S. haematobium* and *S. guineensis* were also detected [8]. Because species classification in our study was based on egg morphology and specimen type without molecular or antigen-based confirmation, the findings represent microscopy-based species distribution among detected infections rather than definitive species prevalence in the study population.

After adjustment for potential confounders, use of an unimproved main water source, use of an unimproved toilet facility and absence of a handwashing facility remained independently associated with microscopically detectable schistosomiasis. This pattern is consistent with the established transmission ecology of schistosomiasis, which involves contact with cercariae-infested freshwater and inadequate sanitation that permits schistosome eggs to contaminate the environment [1,10,16].

The association with an unimproved water source is plausible because women in endemic settings may encounter unsafe freshwater while collecting water, washing clothes or bathing. A systematic review and meta-analysis found that access to safe water was associated with lower odds of schistosomiasis, while studies among pregnant women have linked continued stream use with infection [10,18].

The association with an unimproved toilet facility is also epidemiologically plausible because inadequate sanitation facilitates environmental contamination and continued transmission. This interpretation is supported by the WASH–schistosomiasis meta-analysis by Grimes et al. and WHO guidance that includes sanitation improvement within broader schistosomiasis-control strategies [10,19]. Evidence from Zimbabwe has similarly shown associations between household latrine access, drinking-water source and urogenital schistosomiasis among pregnant women [11].

The absence of a handwashing facility should be interpreted within the broader household WASH context. Handwashing infrastructure does not directly prevent cercarial penetration, but its absence may indicate poorer household water and sanitation conditions. This variable may therefore represent broader environmental deprivation and constrained access to basic hygiene infrastructure rather than an isolated behavioural determinant [10,19].

Education level and body mass index did not retain independent associations after adjustment. In maternal schistosomiasis studies, sociodemographic characteristics may be associated with infection in crude analyses but weaken after adjustment for more proximal or contextual exposures, including water source, sanitation and location-specific transmission risk [8,11,20].

These findings should be interpreted as associations rather than evidence that the measured household WASH conditions directly caused infection. Water source, toilet type and access to a handwashing facility may reflect broader social and environmental circumstances, including socioeconomic position, place of residence, proximity to infested freshwater and unmeasured occupational, domestic or recreational water-contact behaviours. Residual confounding by these factors cannot be excluded.

Women reporting fewer than four ANC contacts had higher adjusted odds of microscopically detectable schistosomiasis than those reporting four to seven contacts. However, evidence directly linking ANC frequency to schistosomiasis remains limited because most studies recruit pregnant women through ANC services without evaluating visit frequency as an independent exposure [21,22]. ANC attendance may reflect healthcare access, socioeconomic position, geographical location, health awareness and freshwater exposure rather than directly influencing infection. The absence of a clear dose–response pattern and the small number of women reporting eight or more contacts require cautious interpretation.

The public-health significance of these findings extends beyond documenting infection prevalence. The observed pattern suggests that microscopically detectable maternal schistosomiasis occurs within broader inequalities in access to safe water, sanitation and hygiene. Prevention may therefore benefit from integrating context-appropriate schistosomiasis risk assessment and health education into maternal health services, together with collaboration between maternal health, WASH and community health programmes in endemic areas. Although this study did not assess subsequent maternal or neonatal outcomes, the detection of infection at delivery indicates that some women may reach childbirth with previously undetected schistosomiasis. Further research is needed to determine how schistosomiasis assessment and prevention can be incorporated effectively into maternal health services.

### Strengths and limitations

This study used standardized questionnaire and laboratory procedures to estimate the prevalence of microscopically detectable schistosomiasis and describe the microscopy-based species distribution among microscopy-positive women delivering at a regional referral hospital. Examination of both stool and urine specimens enabled assessment of intestinal and urogenital schistosomiasis, while duplicate Kato–Katz preparations and independent re-examination of a randomly selected proportion of microscopy preparations strengthened laboratory quality assurance. The final sample exceeded the calculated minimum, and the analysis considered sociodemographic, clinical and household WASH characteristics.

Several limitations should be considered. First, recruitment from a single regional referral hospital limits generalizability. Women delivering at JRRH may differ from those delivering in lower-level facilities, private facilities or at home in terms of obstetric risk, referral status and access to healthcare. Participants’ referral origins were not systematically recorded, preventing comparisons between women from Jinja City and those referred from surrounding districts. The prevalence estimate should therefore be interpreted as the prevalence of microscopically detectable schistosomiasis among women delivering at JRRH rather than as a population prevalence for eastern Uganda. Second, diagnosis was based on one stool and one urine specimen per participant. Because schistosome egg excretion varies within and between days and microscopy has limited sensitivity for low-intensity infections, some infections may have been missed. The observed prevalence may therefore underestimate the underlying burden, and outcome misclassification may have affected the estimated associations. Species classification relied on egg morphology and specimen type without antigen-based or molecular confirmation; consequently, atypical or hybrid forms could not be reliably identified. The reported species distribution should therefore be interpreted as microscopy-based classification among detected infections. Third, the cross-sectional design precluded establishing temporality or causality. Direct freshwater-contact behaviours, including swimming, fishing, bathing, washing clothes, crossing rivers and farming near freshwater, were not comprehensively measured. The analysis could not therefore separate the influence of household WASH infrastructure from direct exposure to potentially infested freshwater. Residual confounding by these behaviours, socioeconomic circumstances and proximity to transmission sites remains possible.

Fourth, only 17 women reported eight or more ANC contacts. Although using four to seven contacts as the reference category provided more interpretable comparisons based on the largest group, the estimate for eight or more contacts remained imprecise, with a wide confidence interval. The ANC findings should therefore be considered exploratory and interpreted cautiously.

Finally, the expanded model contained 13 predictor parameters relative to 44 microscopy-positive outcomes and may have been vulnerable to overfitting and unstable estimates, particularly for categories with few participants. Nevertheless, the principal WASH associations remained consistent in the parsimonious conventional and Firth penalized sensitivity analyses. These sensitivity analyses support the robustness of the principal findings but do not eliminate the possibility of overfitting; therefore, the estimates require cautious interpretation and confirmation in independent studies.

## Conclusions

Microscopically detectable schistosomiasis was present among women delivering at Jinja Regional Referral Hospital, with S. haematobium predominating among microscopy-classified infections. Unimproved household water source, unimproved toilet facility and absence of a handwashing facility remained associated with microscopically detectable schistosomiasis after multivariable adjustment. Fewer than four ANC contacts were also associated with higher odds of microscopy-detected infection than four to seven contacts, although this exploratory finding requires cautious interpretation. These cross-sectional associations do not establish causality. The findings support further evaluation of context-appropriate schistosomiasis risk assessment within maternal health services and reinforce the importance of coordinated WASH and schistosomiasis-control strategies in endemic settings

## Supporting information

S1 Data De-identified dataset used for the analysis. (XLS)

S1 Table Sensitivity analysis of factors associated with microscopically detectable maternal schistosomiasis at delivery. Comparison of expanded and parsimonious conventional logistic regression models with corresponding Firth penalized sensitivity analyses.
(DOCX)

## Abbreviations

CI: Confidence Interval
ELISA: Enzyme-Linked Immunosorbent Assay
HBM: Health Belief Model
HIV: Human Immunodeficiency Virus
JRRH: Jinja Regional Referral Hospital
KIU: Kampala International University
MoH Uganda: Ministry of Health Uganda
NCP: National Control Program
PCR: Polymerase Chain Reaction
REC: Research Ethics Committee
WHO: World Health Organization
