# Supplementary material for: Microscopically detectable maternal schistosomiasis at delivery in Eastern Uganda: Prevalence, microscopy-based species distribution and associated household WASH characteristics in a hospital-based cross-sectional study
Source: PLOS Glob Public Health. 2026 Oct 23;6(10):e0006297. doi: 10.1371/journal.pgph.0006297 (PMC13693890; doi:10.1371/journal.pgph.0006297)
Supplement: Supplementary file 1 [file pgph.0006297.s002.docx]

**S1 Table. Sensitivity analyses of factors associated with microscopically detectable schistosomiasis among women delivering at Jinja Regional Referral Hospital (N = 392)**

| Variable | Category | Expanded conventional model | Expanded Firth model | Parsimonious conventional model | Parsimonious Firth model |
| --- | --- | --- | --- | --- | --- |
| Employment status | Formal employment | 1.00 (Reference) | 1.00 (Reference) | — | — |
|  | Unemployed | 1.71 (0.64–4.59); 0.283 | 1.61 (0.63–4.11); 0.319 | — | — |
|  | Informal employment | 0.55 (0.18–1.71); 0.303 | 0.57 (0.19–1.65); 0.297 | — | — |
| Education level | Tertiary | 1.00 (Reference) | 1.00 (Reference) | — | — |
|  | No formal education | 3.45 (0.95–12.52); 0.059 | 3.11 (0.92–10.50); 0.068 | — | — |
|  | Primary education | 1.72 (0.52–5.67); 0.375 | 1.59 (0.52–4.90); 0.417 | — | — |
|  | Secondary education | 1.65 (0.50–5.48); 0.413 | 1.55 (0.50–4.81); 0.447 | — | — |
| Household water source | Improved | 1.00 (Reference) | 1.00 (Reference) | 1.00 (Reference) | 1.00 (Reference) |
|  | Unimproved | **3.11 (1.46–6.64); 0.003** | **2.88 (1.40–5.92); 0.004** | **2.40 (1.22–4.73); 0.012** | **2.34 (1.20–4.57); 0.012** |
| Household toilet facility | Improved | 1.00 (Reference) | 1.00 (Reference) | 1.00 (Reference) | 1.00 (Reference) |
|  | Unimproved | **4.40 (2.01–9.67); <0.001** | **3.97 (1.89–8.33); <0.001** | **3.96 (1.94–8.08); <0.001** | **3.82 (1.90–7.68); <0.001** |
| Handwashing facility | Available | 1.00 (Reference) | 1.00 (Reference) | 1.00 (Reference) | 1.00 (Reference) |
|  | Unavailable | **2.12 (1.02–4.41); 0.043** | **2.03 (1.01–4.07); 0.048** | **2.35 (1.22–4.54); 0.011** | **2.33 (1.22–4.45); 0.011** |
| Number of ANC contacts | **4–7 contacts** | **1.00 (Reference)** | **1.00 (Reference)** | — | — |
|  | <4 contacts | **8.43 (3.21–22.18); <0.001** | **7.12 (2.93–17.31); <0.001** | — | — |
|  | ≥8 contacts | 4.13 (0.67–25.43); 0.126 | 4.19 (0.79–22.26); 0.093 | — | — |
| Body mass index | Normal weight | 1.00 (Reference) | 1.00 (Reference) | — | — |
|  | Underweight | 5.46 (0.89–33.55); 0.067 | 5.34 (0.96–29.73); 0.056 | — | — |
|  | Overweight | 0.79 (0.36–1.72); 0.548 | 0.80 (0.38–1.69); 0.558 | — | — |
|  | Obese | 2.73 (0.85–8.73); 0.091 | 2.62 (0.85–8.09); 0.093 | — | — |

*Values are adjusted odds ratios (aORs) with 95% confidence intervals (CIs). The parsimonious models included the three household WASH variables. An em dash indicates that the variable was not included. ANC, antenatal care; CI, confidence*
